# Neural oscillation of single silicon nanowire neuron device with no external bias voltage

**DOI:** 10.1038/s41598-022-07374-2

**Published:** 2022-03-03

**Authors:** Sola Woo, Sangsig Kim

**Affiliations:** Department of Electrical Engineering, Korea University, 145 Anam-ro, Seongbuk-gu, Seoul, 02841 Republic of Korea

**Keywords:** Nanoscience and technology, Nanoscale devices, Electronic devices

## Abstract

In this study, we perform simulations to demonstrate neural oscillations in a single silicon nanowire neuron device comprising a gated *p–n–p–n* diode structure with no external bias lines. The neuron device emulates a biological neuron using interlinked positive and negative feedback loops, enabling neural oscillations with a high firing frequency of ~ 8 MHz and a low energy consumption of ~ 4.5 × 10^−15^ J. The neuron device provides a high integration density and low energy consumption for neuromorphic hardware. The periodic and aperiodic patterns of the neural oscillations depend on the amplitudes of the analog and digital input signals. Furthermore, the device characteristics, energy band diagram, and leaky integrate-and-fire operation of the neuron device are discussed.

## Introduction

Neuromorphic computation inspired by human brain architecture has great potential to overcome technical challenges in centralized and sequential computation based on the von Neumann architecture^1–3^. The von Neumann architecture requires intrinsically separate physical units for computing (a central processing unit) and memory (static and dynamic random access memory), necessitating data to be carried back and forth between them through bus lines^4^. Busy bus lines cause a bottleneck in data transfer, resulting in a significant decrease in computing speed and a substantial increase in power consumption^4^. In contrast, biological neurons perform parallel, distributed, and event-driven computations, enabling energy-efficient operation^5^. Hence, neuromorphic computing aims to emulate the data processing paradigm found in the topology of the biological brain which can pave the way for massively parallel information processing with extremely low power consumption^6,7^.

In neuromorphic computation driven by spiking neural networks, an artificial neuron is an integral component that interlinks synapses, thus promoting fast and energy-efficient information processing. Emulation of neuron information transfer in biological signaling systems requires positive and negative feedback loops as common regulatory elements^8–10^. However, most artificial neurons demand dozens of transistors to emulate biological neuron operation, in turn, greatly sacrificing the advances in integration density and power consumption^4,11–13^. To improve the integration capabilities, diverse neuron devices and circuits have been widely researched: NPN devices with double gates on a silicon-on-insulator (SOI)^14^, feedback field-effect transistors (FBFETs)^15–17^, skyrmion devices based on magnetic tunnel junction^18^, resistive random access memory (ReRAM)^19^, conductive bridge random access memory (CBRAM)^20^, ferroelectric field-effect transistors (FeFET)^21,22^ and phase-change devices^23^. However, these neuron devices and circuits require numerous component transistors and consume considerable energy to operate in addition to external bias voltages necessary for tuning firing voltages. Moreover, the skyrmion is incompatible with CMOS technology, and the phase-change devices have reliability issues due to their harsh operating conditions^24^. Such devices also consume standby power constantly in the form of external bias voltages while anticipating signal application. Therefore, a single artificial neuron device without consistent power consumption is much more desirable as it can resolve both integration density and power consumption issues. In this paper, we propose a single silicon nanowire neuron device with no external bias voltage capable of normally-off integrate-and-fire operation technology, which achieves zero standby power.

## Methods

### Device structure and simulation

**Figure 1:**
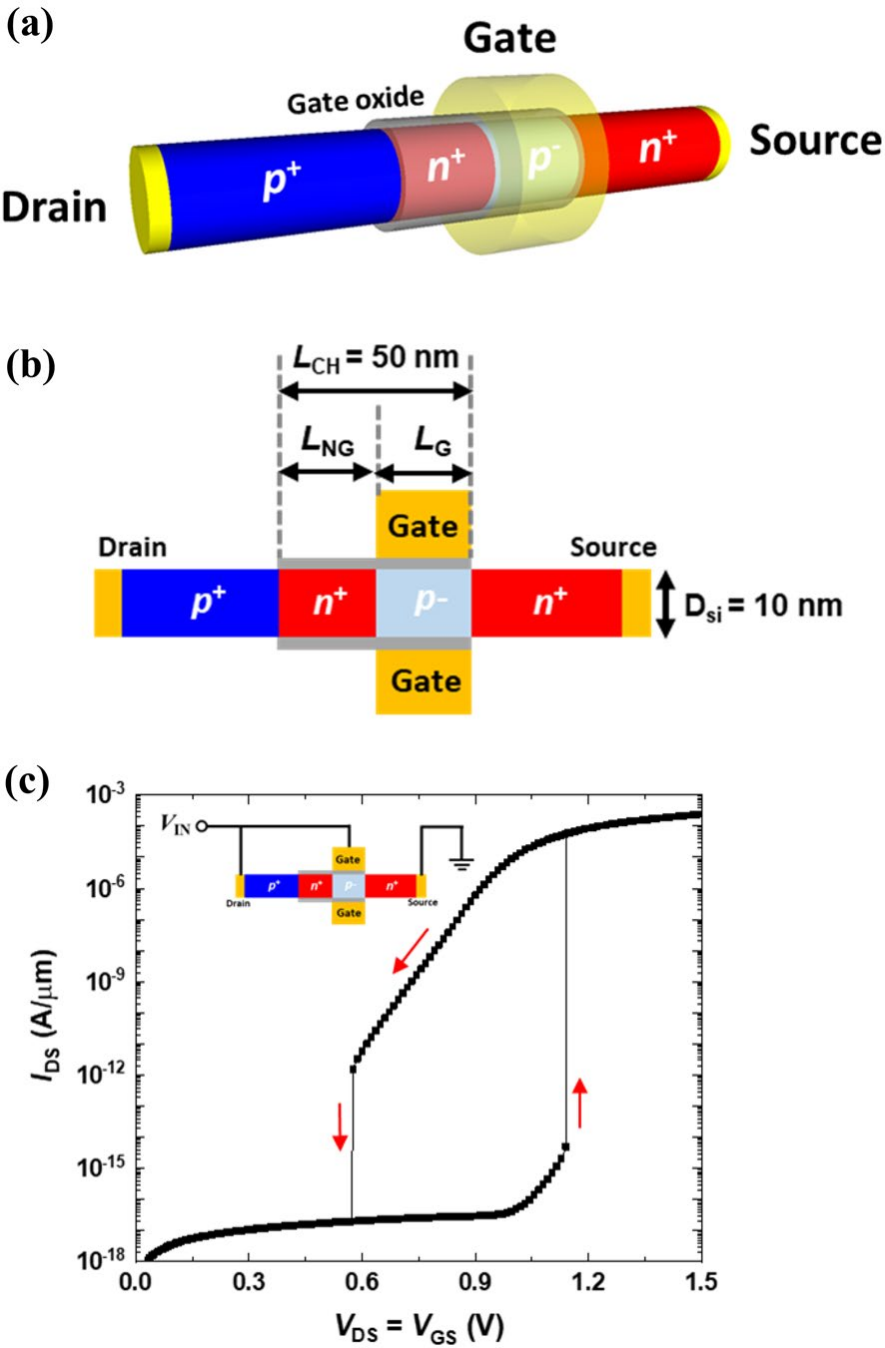
(**a**) Three-dimensional structure and (**b**) cross-sectional view of a single silicon nanowire-based neuron device. (**c**) *I*_DS_–*V*_DS_ output characteristics of the single silicon nanowire neuron device with connected gate and drain terminals.

Figure 1a, b show, respectively, a schematic and cross-sectional view of a single silicon nanowire neuron device with a gated *p-n-p-n* diode structure. The dimensional parameters are channel length (*L*_CH_), gated channel length (*L*_G_), non-gated channel length (*L*_NG_), channel diameter (*D*_Si_), and gate oxide thickness (*T*_OX_) with values 50, 25, 25, 10, and 2 nm, respectively. The doping concentrations of the p-type drain, n-type non-gated channel, and *n*-type source regions were 5 × 10^19^ cm^−3^, and the gated channel region was lightly *p*-type doped (2 × 10^15^ cm^−3^). The device characteristics, energy band diagrams, and leaky integrate-and-fire (LIF) operations were investigated using a commercial TCAD simulation software (Synopsys Sentaurus, version O-2018.06-SP1)^25^. Fundamental device models were included in our TCAD simulations, including Fermi statistics model, bandgap narrowing model, carrier-carrier scattering, Shockey–Read–Hall recombination, and Auger recombination. Moreover, density gradient quantum potential model was used to treat the quantum effect occurring in ultrathin silicon nanowires^25^.

The single silicon nanowire neuron device operates as a two-terminal device with connected gate and drain terminals to accelerate the function of neural oscillations via interlinked positive and negative feedback loops. The *I*_DS_–*V*_DS_ output characteristics from the simulation are shown in Fig. 1c. As the connected gate and drain voltage (input voltage) is swept forward from 0.00 to 1.50 V, the formation of a positive feedback loop triggers an abrupt increase in the drain current at 1.14 V. During the reverse bias sweep, the generation of a negative feedback loop leads to an abrupt decrease in the drain current at 0.57 V. In our neuron device, the positive and negative feedback loops generate neural oscillations, with the former causing a latch-up phenomenon, while the latter, a latch-down phenomenon. In the next section, we discuss the device characteristics and operating principles of this neuron device. Further, we analyze in detail the LIF operations of the interlinked positive and negative feedback loops occurring in the device.

## Result and discussion

### Device characteristics and operating principle

**Figure 2:**
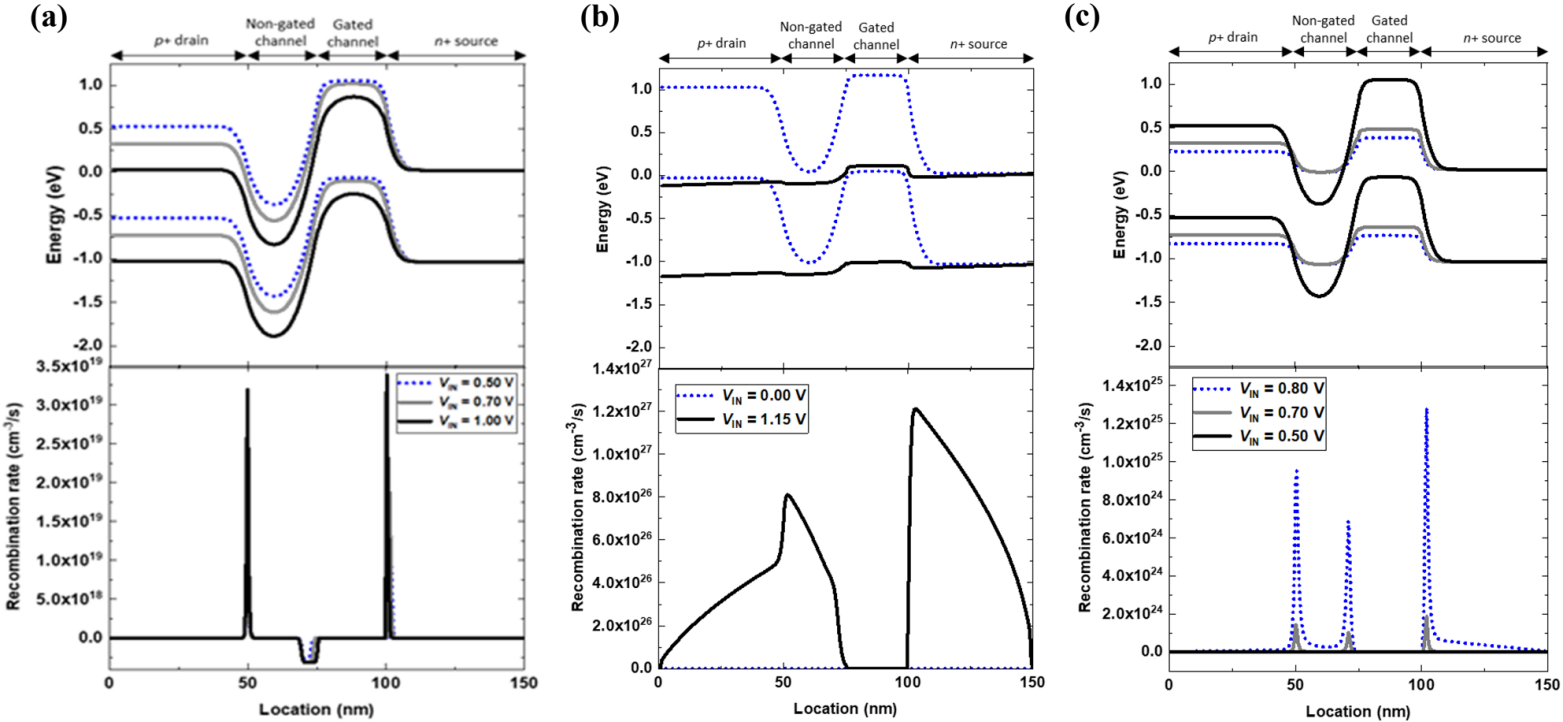
Energy band diagrams and recombination rates of the single silicon nanowire neuron device during its integrate-and-fire and reset responses: (**a**) leaky integration states at *V*_IN_ = 0.50, 0.70, and 1.00 V during forward sweep, (**b**) firing state at *V*_IN_ = 1.15 V, and (**c**) reset states at *V*_IN_ = 0.80, 0.70, and 0.50 V during reverse sweep.

The fundamental operation of a single silicon nanowire neuron device is based on positive and negative feedback loops in the gated *p-n-p-n* diode structure. Figure 2 shows the energy band diagrams and recombination rates of the neuron device; these are associated with the *I*_DS_–*V*_DS_ output characteristics (Fig. 1c). The input voltage (*V*_IN_) ranging from 0.00 to 1.00 V was applied to the connected gate and drain terminals for a leaky integration in the neuron device before triggering the positive feedback loop. The effect of the input voltage is divided into the gate voltage and drain voltage functions regarding the positive and negative feedback loops in the gated *p-n-p-n* diode structure. First, as the drain voltage increases in the gated *p-n-p-n* diode structure, both the drain and non-gated channel junction and the gated channel and source junction are forward biased, whereas the junction of non-gated and gated channels is more significantly reverse biased. At the drain and non-gated channel junction (the gated channel and source junction), electron–hole recombination increases from 1.3 × 10^19^ (9.4 × 10^18^) to 3.2 × 10^19^ (1.5 × 10^19^), lowering the height of the junction potential barrier. At the junction of non-gated and gated channels, electron–hole generation is increased by extension (≈ 4 nm) of the depletion region, as depicted in the bottom panel of Fig. 2a. The generated charge carriers accumulate in the potential wells of the non-gated and gated channels—electrons in the conduction band of the former, holes in the valence band of the latter (top panel of Fig. 2a). Second, as the gate voltage increases from 0.00 to 1.00 V in the gated *p-n-p-n* diode structure, the conduction band (the valence band) edge in the gated channel region is lowered from 1.03 to 0.37 eV (from − 0.02 to − 0.69 eV), allowing the injection of electrons into the potential well in the conduction band of the non-gated channel region^26^. The potential barrier modulation by the input voltage stimulates the electron injection from the source to the channel region; the injected electrons accumulate in the potential well in the non-gated channel region. The electron accumulation lowers the height of the potential barrier, which allows holes to be injected. The injected holes accumulate and encourage the injection of more electrons. The resulting accumulation of charge carriers induced by the input voltage emulate the leaky integration of biological systems.

As the input voltage exceeds the threshold voltage of 1.14 V, the lowering of the potential barrier by the accumulated charge carriers activates the positive feedback loop. The energy band diagram at *V*_IN_ = 1.15 V (top panel of Fig. 2b) corresponds to the firing state. The positive feedback loop eliminates the potential well, and thereby, electron–hole recombination occurs in the entire region of the gated *p-n-p-n* diode structure (bottom panel of Fig. 2b). After the neuron device fires, the input voltage decreases from 1.15 to 0.70 V; nevertheless, the positive feedback loop persists until the negative feedback loop is initiated. The potential barrier modulation by the reduction of the input voltage stimulates the electron–hole recombination at the drain and non-gated junction, non-gated channel and gated channel junction, and gated-channel and source junction; the electron–hole recombination gradually regenerates a potential barrier in each junction, which impedes electrons to be injected. The charge carrier recombination increases the potential barrier height, blocking the electron injection. The mutual interaction between the potential barriers and charge carriers produces the negative feedback loop, which leads to the neuron device in the latch-down state. As the input voltage drops below the latch-down voltage, the potential barrier is regenerated by the negative feedback loop, as shown in the top panel of Fig. 2c. In contrast to the positive feedback loop which is generated by accumulated charge carriers in the potential well, the negative feedback loop removes the accumulated charge carriers, which is caused by electron–hole recombination at the junctions of non-gated and gated channels, as shown in the bottom panel of Fig. 2c.

### Neural oscillation of single silicon nanowire neuron device

**Figure 3:**
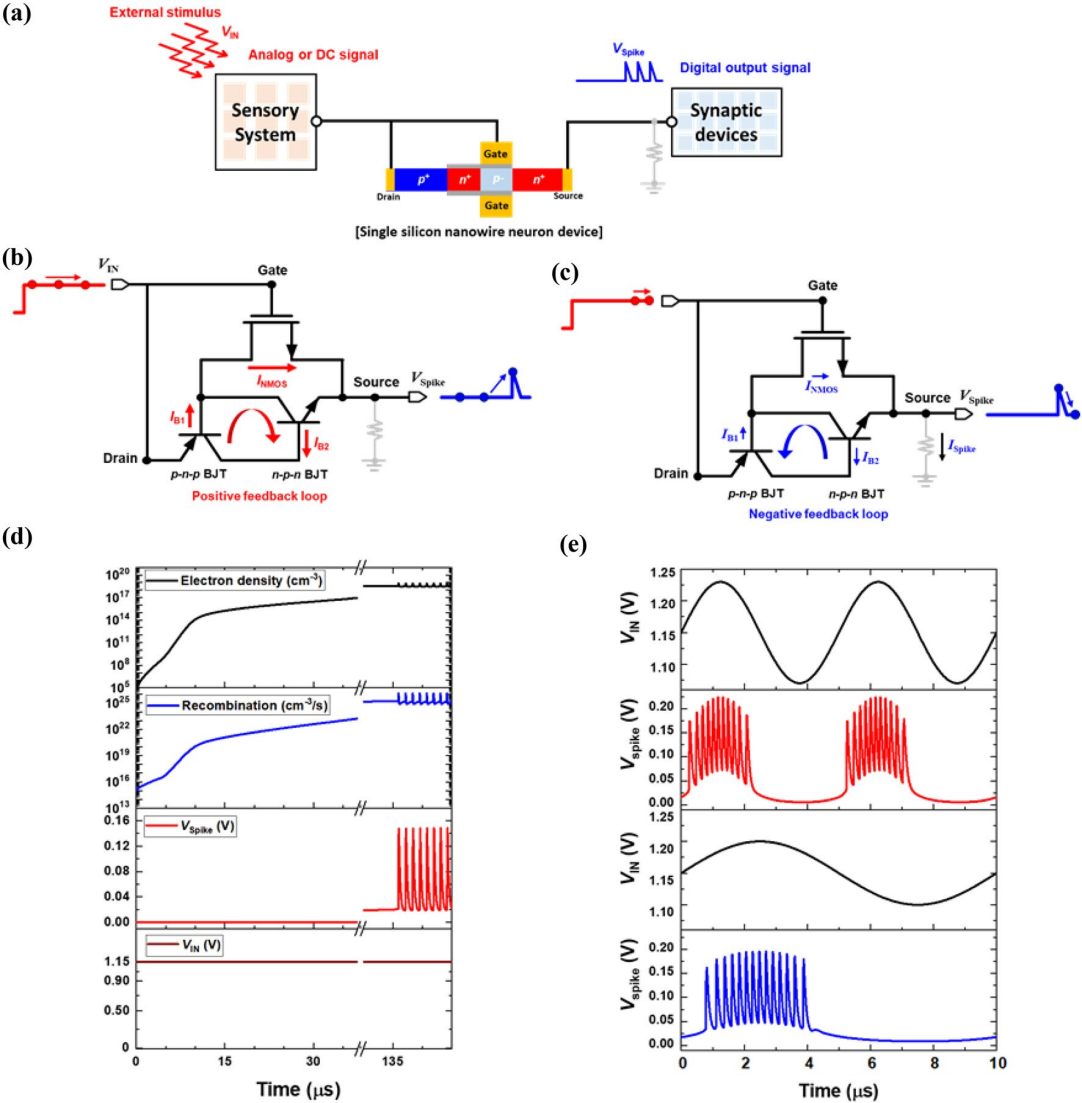
(**a**) Block diagram of front-end neuromorphic hardware system, consisting of sensory system, synaptic devices, and neuron device, receiving external stimulus. (**b**) Spike and (**c**) reset mechanism in an equivalent circuit of the single silicon nanowire neuron device, and (**d**) electron density in the non-gated channel region, recombination rate in the non-gated channel region, spike voltage, and input voltage as functions of time. (**e**) A spike response of the neuron device to sinusoidal analog input signals.

The dynamics of neural oscillation in a single silicon nanowire neuron device using interlinked positive and negative feedback loops were investigated by applying analog and digital input signals. Figure 3a shows a neuromorphic block diagram composed of a sensory system, synaptic devices, and a single silicon nanowire neuron device. The sensory system perceives an external stimulus and transmits analog AC and DC signals into the neuron device. Subsequently, the neuron device fires spike voltages (*V*_Spike_) toward the synapse devices. The spike and reset mechanisms of the neuron device enabling information transfer for synapse devices are explained using an equivalent circuit. The equivalent circuit of our neuron device consists of coupled bipolar junction transistors (BJTs) and an n-channel metal-oxide- semiconductor field-effect transistor (MOSFET)^27^, as shown in Fig. 3b, c. The input voltage, output spike voltage (*V*_Spike_), recombination rate, and electron density during the LIF operation are plotted as functions of time in Fig. 3d. When a constant DC input voltage of 1.15 V is applied to the input voltage node (*V*_IN_), the *n*-channel MOSFET is partially turned on and the current component *I*_NMOS_ is increased. Simultaneously, the electron density in the potential well of the non-gated region is increased because of an increase in the emitter current component *I*_B1_ in the *p–n–p* BJT and collector current component *I*_B2_ in the *n–p–n* BJT. Moreover, the recombination rate in the non-gated channel region increases with time owing to the accumulation of electrons in the potential well. When the positive feedback loop is initiated by accumulated charge carriers, spiking behavior with oscillations are generated by the latch-up phenomenon. After the generation of the positive feedback loop, as the voltage across the neuron device decreases with an increase in the source node voltage (*V*_Spike_), which is caused by the abrupt increase in the current, the accumulated charge carriers are removed. This phenomenon is the latch-down state, which is enabled by the negative feedback loop. After an effective refractory period, neural oscillations of the neuron device were present in the *V*_Spike_ as a function of time. Following the spiking behavior in the neuron device induced by the positive feedback loop, the output spike voltage (*V*_Spike_) automatically returns to the initial state, which corresponds to the reset mechanism induced by the negative feedback loop.

Our neuron device produces digital output signals with neural oscillations for DC as well as analog AC input signals. Sinusoidal analog input signals, in which dynamic neural oscillations can be implemented using positive and negative feedback loops, were applied to the neuron device. When the *V*_IN_ is above 1.15 V in the sinusoidal analog input signal, the neuron device produces neural oscillations (Fig. 3e). As the amplitude and frequency of the sinusoidal analog input signal range from 100 to 50 mV and 200 to 100 kHz, respectively, the number of spike events per time can be tuned by adjusting the input signal. Therefore, the modulation of the neural oscillation frequency is enabled by varying the amplitude, offset voltage, and period of the sinusoidal input signals.

**Figure 4:**
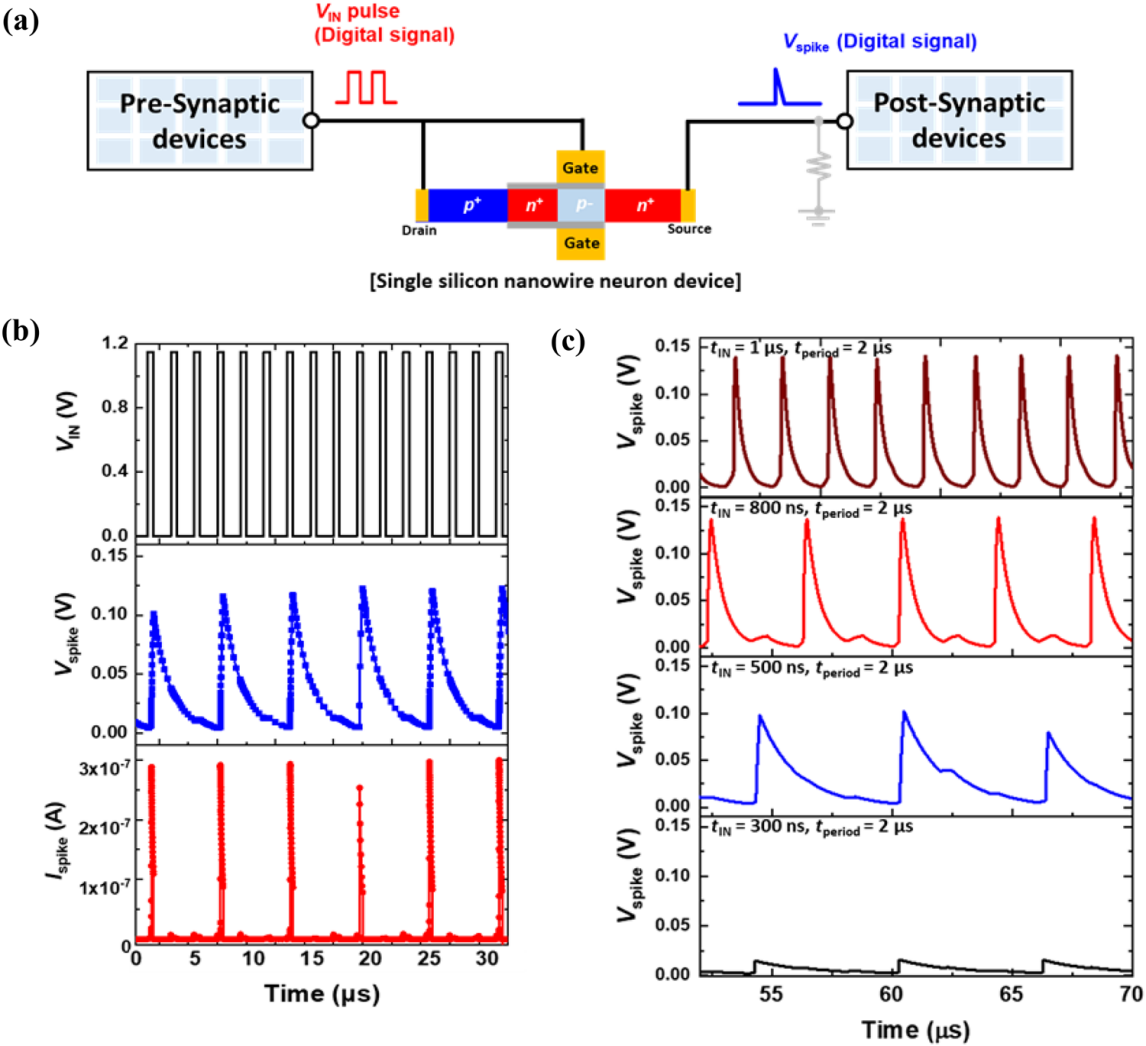
(**a**) Block diagram of artificial neuron device, pre- and post- synaptic devices constituting the neuromorphic hardware system. (**b**) Input voltage, spike voltage, and spike current as functions of time. (**c**) A spike response of the single silicon nanowire neuron device for time widths of input voltage pulse (*t*_IN_) of 1 μs, 800 ns,500 ns, and 300 ns.

The neuron device can deliver digital signals from pre-synaptic devices to post-synaptic devices (Fig. 4a). Figure 4b shows the input voltage (*V*_IN_), output spike voltage (*V*_spike_), and output spike current (*I*_spike_) as functions of time. The integration and firing operations are performed in the neuron device for the transmission of *V*_IN_ pulses (with 1.15 V, a time width of 500 ns, and a period of 2 μs). As the *V*_IN_ pulses are input to the neuron device, charge carriers constituting the *V*_IN_ pulses accumulate (or integrate) in the potential well of the neuron device. The lowering of the potential barrier height by repeated VIN pulses initiates a positive feedback loop in the neuron device. Then, the generated *V*_spike_ (*I*_spike_) rises rapidly from 0.00 V (2.6 × 10^−10^ A) to 0.14 V (2.9 × 10^−7^ A), which corresponds to the firing of *V*_spike_ (*I*_spike_). In the reset stage, *V*_spike_ (*I*_spike_) returns to the initial voltage (current) through a negative feedback loop. Thus, our neuron device effects periodic spike and reset operations on *V*_IN_ pulses. Moreover, the firing frequency and *V*_spike_ can vary with the time width of the input voltage (*t*_IN_). As *t*_IN_ decreases from 1 μs to 300 ns, the number of spikes within a defined time (20 μs) decreases from 9 to 3, while the firing frequency and *V*_spike_ decrease from 507 to 167 kHz and from 0.140 to 0.015 V, respectively (Fig. 4c). Because of the shorter *t*_IN_, fewer charge carriers integrate in the potential well of the neuron device, which in turn calls for a greater number of *V*_IN_ pulses for integration and firing operations.

**Figure 5:**
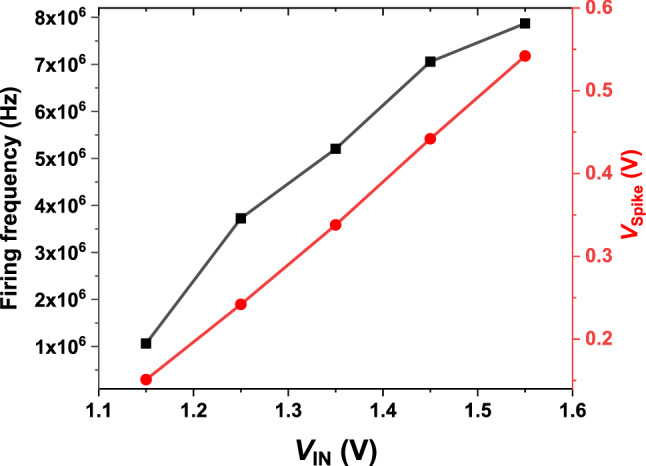
Firing frequency and spike voltage as functions of the amplitude of the constant DC input voltage.

The firing frequency and *V*_spike_ of our neuron device depend on the amplitude of *V*_IN_ (Fig. 5). As *V*_IN_ increases from 1.15 to 1.55 V, more charge carriers accumulate in the potential well leading to decrease in the time for triggering the positive feedback loop. Consequently, the firing frequency (*V*_spike_) increases from 1.1 MHz (0.15 V) to 7.8 MHz (0.54 V). The adjustment of the amplitude of VIN enables our neuron device to control the firing frequency and *V*_spike_. It should be noted that the firing frequency saturates at ~ 8 MHz despite the increment in *V*_IN_. This is caused by the inner capacitance components in our neuron device giving rise to RC delay.

In Table 1, our neuron device is compared with other neuron devices and circuits in terms of operating mechanism, number of components, number of external bias lines, supply voltage, energy consumption, and firing frequency. External bias lines are indispensable for tuning spiking voltages in neuron devices with independent double-gate field-effect transistors^14^; such devices have high energy consumption (9.5 × 10^−13^ J) and a low firing frequency (~ 300 Hz). Neuron circuits using a positive feedback mechanism^15–17^ reported by other research groups require more than five components and two external bias lines to implement their integration and firing operations. In addition, these neuron circuits^15,16^ consume substantial energy (2.5 × 10^−13^ J and 6.2 × 10^−13^ J) while having low firing frequencies (~ 300 Hz and ~ 30 kHz). Although our previous neuron circuit using a positive feedback mechanism^28^ had an energy consumption (2.9 × 10^−15^ J) lower than our present neuron device (4.5 × 10^−15^ J), the latter had a large area occupied by a membrane capacitor and needs one external bias line. The partially depleted SOI MOSFET neuron device based on the band-to-band tunneling mechanism requires an external reset circuit and a threshold detector circuit^24^, even though it has a relatively low energy consumption. As for capacitor-less ReRAM and PCRAM^19^, their neuron operation should need more than ten transistors with several external bias voltages. In the neuron operation of FeFETs^21,22^, 1 nF and 1 pF capacitors should be used as the membrane, making the neuron hard to achieve high integration density. Among these neuron devices and circuits, our neuron device using a gated p–n–p–n diode structure is the most energy-efficient owing to its low energy consumption (4.5 × 10^−15^ J), high firing frequency (~ 8 MHz), and absence of external bias voltage; note that neuron devices and circuits including our neuron device require peripheral circuits receiving synaptic pulses and delivering firing voltages. Our neuron device can implement a neuromorphic system by bidirectionally connecting with transposable synapstic SRAM using feedback field-effect transistor, and enables fast information transfer due to its high firing frequency^30^. Furthermore, our neuron device that is in normally-off state until synaptic pulses (analog and digital input signals) reach this device achieves zero standby power without external bias voltage.

**Table 1:** Comparison of performance parameters between the neuron device of the present study and the neuron devices and circuits developed by other research groups.

References	Based device (length)	Approximated components	Number of external bias voltage	Supply voltage (V)	Synaptic input type	Energy (J/Spike)	Firing frequency
^14^	MOSFET (500 nm)	1 transistor	1	1.00	Current	9.5 × 10^−13^	~ 300 Hz
^15^	FBFET (1000 nm)	5 transistors	2	1.50, 1.00	Voltage	2.5 × 10^−13^	~ 300 Hz
^16^	FBFET (2000 nm)	6 transistors	2	1.50, 1.10	Voltage	6.2 × 10^−13^	~ 30 kHz
^17^	FBFET (700 nm)	6 transistors	2	1.20, 1.00	Voltage	-	~ 2 MHz
^19^	ReRAM (65 nm)	More than 20 transistors	6	1.30, 1.30 1.30, 1.00 0.50, 1.30	Voltage	2.14 × 10^−12^	~ 10 kHz
^20^	CBRAM (120 nm)	4 transistors 1 capacitor	1	-	Current	5.5 × 10^−10^	~ 1 MHz
^21^	FeFET (50 nm)	4 transistors 1 capacitor	3	0.70, -0.30 -0.30	Voltage	1 × 10^−13^	~ 1 MHz
^22^	FeFET (14 nm)	2 transistors 1 capacitor	2	1.20, 0.30	Voltage	6.3 × 10^−10^	~ 10 kHz
^28^	FBFET (100 nm)	4 transistors 1 capacitor	1	0.27	Current	2.9 × 10^−15^	~ 20 kHz
^29^	TFET (32 nm)	1 transistor	1	-	Voltage	3.2 × 10^−15^	150 kHz
This work	Gated diode (50 nm)	1 gated-diode	0	None	Voltage	4.5 × 10^−15^	~ 8 MHz

## Conclusion

In summary, our single silicon nanowire neuron device with a gated p–n–p–n structure emulates biological neurons using interlinked positive and negative feedback loops. The neuron device produces dynamic neural oscillations in its leaky integrate-and-fire operation. Moreover, the neuron device provides a high firing frequency and low energy consumption without the need for an external bias voltage. The neuron device that acts as a normally-off IF system achieves zero standby power. The high integration capability and remarkable energy efficiency mean that this neuron device can contribute to the achievement of hardware-based spiking neural networks in state-of-the-art neuromorphic architectures.
